# Coinfection of Severe Acute Respiratory Syndrome Coronavirus 2 (SARS-CoV-2) and Bordetella bronchiseptica Pneumonia in a Renal Transplant Patient

**DOI:** 10.7759/cureus.13113

**Published:** 2021-02-03

**Authors:** Sandhya Nagarakanti, Eliahu Bishburg

**Affiliations:** 1 Internal Medicine/Infectious Disease, Newark Beth Israel Medical Center, Newark, USA

**Keywords:** bordetella, covid-19, macrolide

## Abstract

*Bordetella *species cause respiratory infections in both humans and animals. *Bordetella bronchiseptica *(*B. bronchiseptica*) infection is an uncommon pathogen in humans. The clinical spectrum of infections with SARS-CoV-2 includes viral pneumonia of variable severity, with some patients developing acute respiratory distress syndrome (ARDS), requiring mechanical ventilation support. Transplant patients with coronavirus disease (COVID-19) infection have high mortality. Bacterial coinfection, including pneumonia, have been described in patients with COVID-19. We present a renal transplant patient with COVID-19 pneumonia who developed *B. bronchiseptica *superinfection and had a rapid clinical and radiological response to azithromycin treatment.

## Introduction

*Bordetella* species respiratory infections are well known in both humans and animals. The most prevalent* Bordetella* subspecies in humans is *Bordetella pertussis (B. pertussis)*, while in animals it is *Bordetella bronchiseptica *(*B. bronchiseptica*) [1].* B. bronchiseptica* is a zoonotic organism, rarely described as the cause of infection in humans [2]. In humans, infections range from simple upper respiratory tract infection to severe sepsis [2]. Few cases have been described in transplant recipients. We present a renal transplant patient with coronavirus disease (COVID-19) pneumonia who developed a superinfection with *B. bronchiseptica.* To our knowledge, this is the first case of pneumonia with this organism reported in association with COVID-19.

## Case presentation

A 48-year-old male patient was admitted with shortness of breath, fever, generalized malaise and worsening productive cough starting a week prior to admission. The patient’s past medical history was significant for chronic obstructive pulmonary disease, history of renal transplant in 2001, hypertension, diabetes mellitus, obesity, gout, and obstructive sleep apnea. His past surgical history was significant for amputation of the left fourth and fifth toes, and right nephrectomy in 2019. His immunosuppressive regimen consisted of mycophenolate mofetil 500 mg twice daily and prednisone 5 mg per day. He denied any exposure to pets.

On physical examination, the patient's weight was 123 kg with a BMI of 34.4, temperature 99.2 °F, blood pressure 116/56 mm/hg, heart rate 91 beats/minute. Oxygen (O2) saturation was 60% in room air. He was alert and oriented on arrival. Cardiac exam revealed regular heart sounds without murmurs. On lung exam, there were bibasilar crackles. The rest of his physical exam was normal.

White blood cell count was 7.7x10^3/µL ( 4-10.5^3/µL), 29% lymphocytes. Creatinine was 6.6 mg/dL (0.5-1.2 mg/dL) with a glomerular filtration rate (GFR) of 11 mL/min/1.73 m2 and serum glucose 151 mg/dL (range 65-109 mg/dL). His COVID-19 reverse transcription polymerase chain reaction (RT-PCR) test from nasopharyngeal swab was positive. Chest X-ray showed bilateral diffuse consolidations.

**Figure 1:**
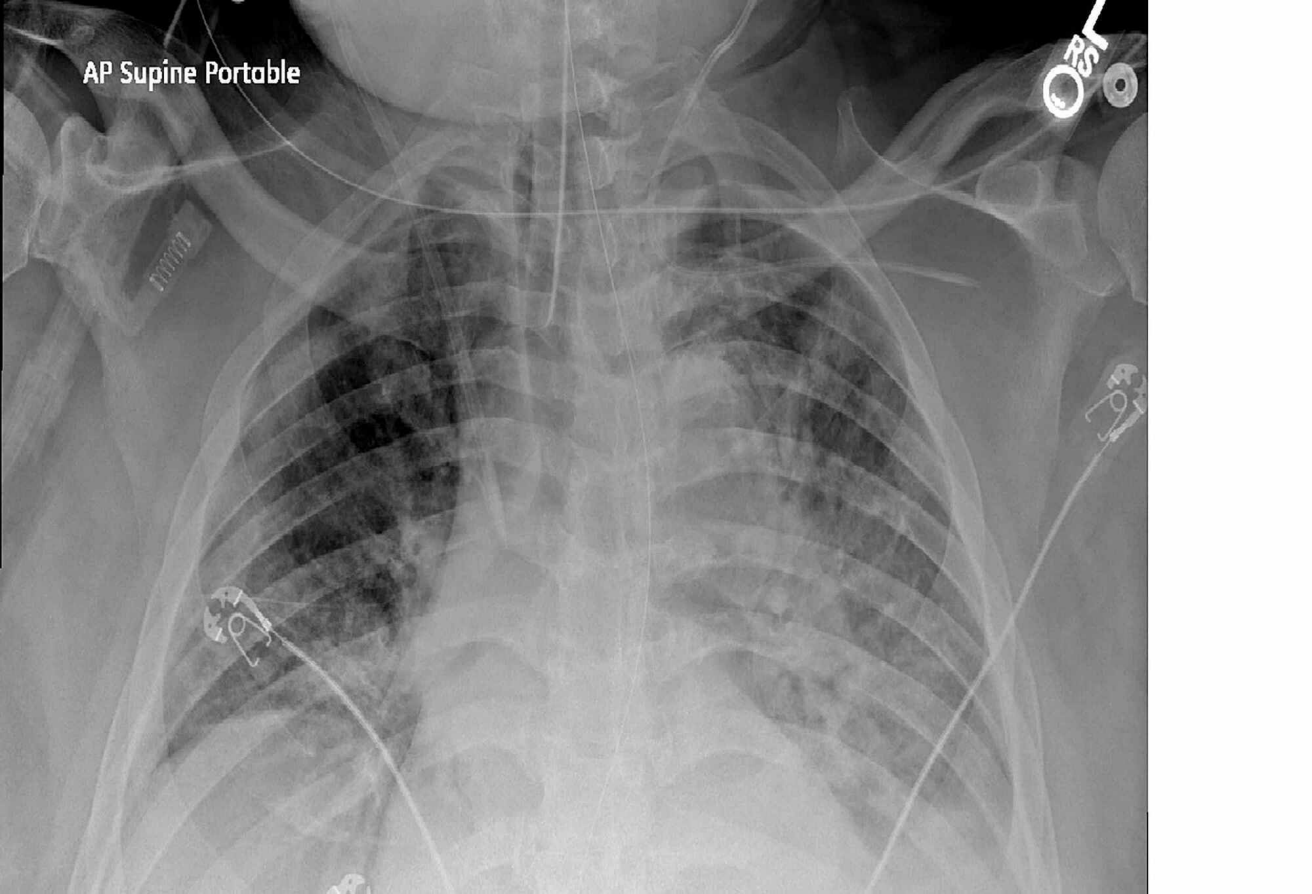
Chest X-ray showing bilateral infiltrates

The patient was initially placed on a 100% non-rebreathing mask and later on a high flow O2 nasal cannula. The patient remained hypoxemic and developed respiratory failure. He was intubated and required mechanical ventilator support. The patient was also initiated on continuous renal replacement therapy. He was started on intravenous ceftriaxone 1 gm and oral azithromycin as well as a course of hydroxychloroquine 400 mg twice daily followed by 400 mg once daily. In addition, he was given intravenous methylprednisolone 100 mg daily. The patient completed five days of treatment of intravenous ceftriaxone and oral azithromycin with an improvement of radiologic findings. After a week, the patient started developing fevers up to 103 °F. Chest X-ray showed an increase in lung infiltrates (Figure 1).

**Figure 2:**
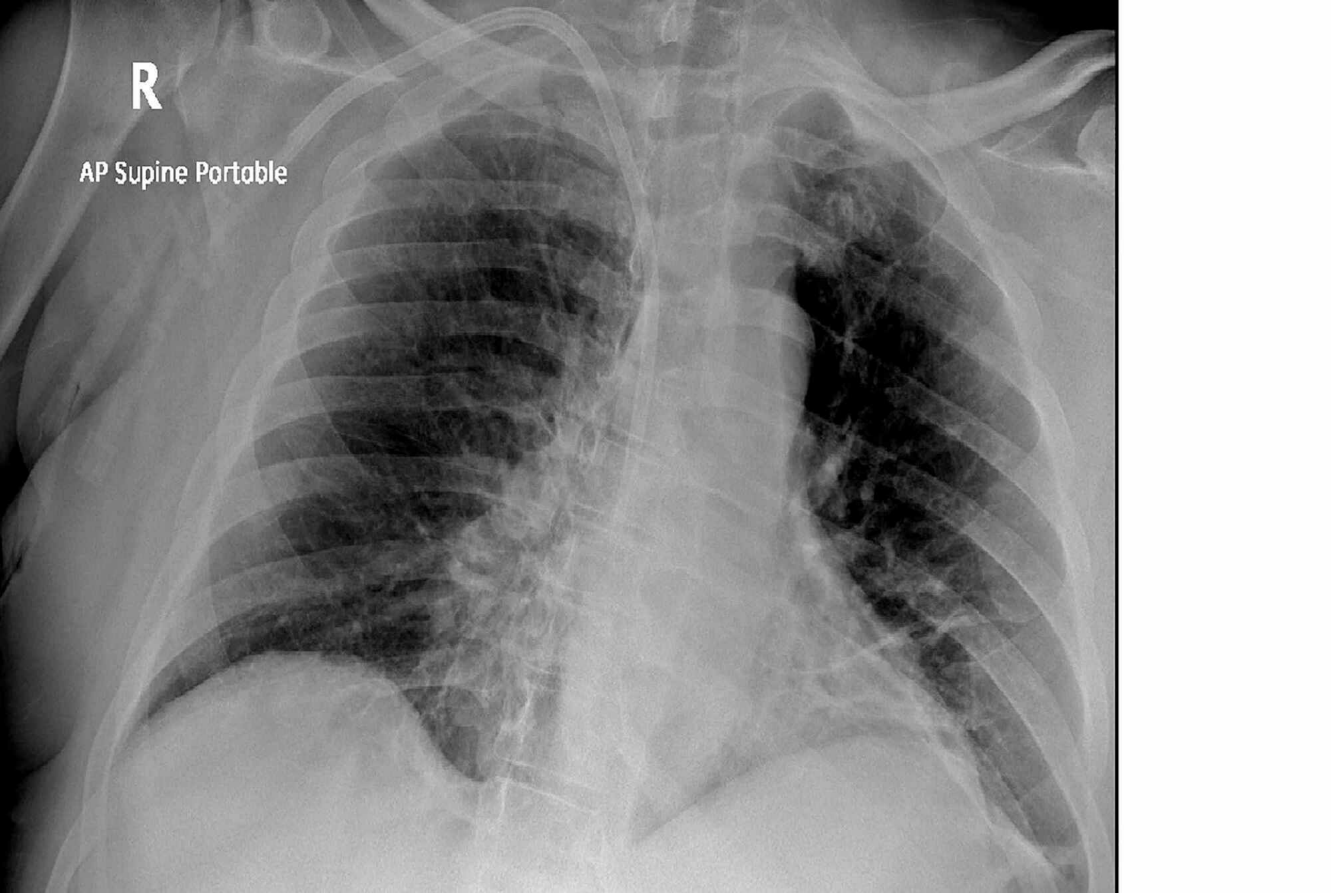
Chest X-ray showing improvement in infiltrates after treatment with azithromycin

Intravenous cefepime 1 gm loading followed by 500 mg next day was given. The patient remained intubated, seemed to be clinically deteriorating, and continued to be febrile, and therefore, intravenous azithromycin 500 mg was given. The sputum sample sent 12 days after intubation grew *B. bronchiseptica*; blood cultures remained negative. The patient became afebrile and was extubated within 24 hours after starting intravenous azithromycin. Intravenous cefepime was stopped and intravenous piperacillin/tazobactam 2.25 gm every eight hours was started and the patient continued with intravenous azithromycin. The organism was resistant to cefepime, intermediately sensitive to ceftazidime, and sensitive to levofloxacin, trimethoprim-sulfamethoxazole, tigecycline, gentamicin, tobramycin, meropenem, and piperacillin/tazobactam. Due to the rapid response to azithromycin leading to extubation, a decision was made to discontinue piperacillin/tazobactam and to continue with azithromycin. The patient continued to improve clinically and remained afebrile. Treatment continued with intravenous azithromycin for a total of 10 days. Chest X-ray two weeks post-discharge showed pulmonary vascular congestion, left lung atelectasis, and resolution of his lung infiltrates (Figure 2). Two months after discharge, the patient was doing well clinically but required intermittent renal replacement therapy.

## Discussion

Nine different subspecies of* Bordetella* have been identified. *B. pertussis* and *B. parapertussis* are most commonly associated with human infections. Clinically, human infections consist of upper respiratory tract infection, pneumonia, endocarditis, peritonitis, meningitis, and bacteremia [2].

*B. bronchiseptica* is known to cause upper respiratory tract infections in dogs, cats, rabbits, swine, and guinea pigs. The organism is the causative agent of kennel cough in dogs and atrophic rhinitis in swine. * B. bronchiseptica* possesses genes similar to *B. pertussis,* suggesting a close relationship between them. The organism can cause serious infection in immunocompromised patients such as HIV-infected patients [3], acute leukemia [4], bone marrow transplant [5], and other malignancies. Patients infected with this organism often report contact with animals prior to the development of infection [6,7]. 

In the recent pandemic of COVID-19, patients with underlying diabetes mellitus, obesity, and immunocompromised patients are at an increased risk of developing severe disease. Patients with a history of solid organ transplantation on immunosuppression have been described with significantly higher mortality [8]. The spectrum of clinical disease with COVID-19 varies from a mild respiratory illness to fulminant pneumonia with ARDS requiring mechanical ventilation. Some patients present with a clinical picture as well as radiological appearance similar to bacterial or atypical pneumonia. In hospitalized patients, it is sometimes difficult to distinguish between SARS-CoV-2 pneumonia and hospital or ventilator-associated pneumonia. Many of these patients are therefore started on antibiotic treatment and are worked up for bacterial or atypical pneumonia. In a recent review, Rawson et al. [9] reviewed the current literature on evidence of bacterial/fungal coinfection in hospitalized COVID-19 patients. They found 62/806 (8%) coinfection with bacterial/fungal organisms. Zhou et al. [8] reported a 15% (28/191) secondary bacterial infection, of which 27 patients died. Reports on atypical organisms infecting COVID-19 patients are lacking, and to our knowledge, there is no report of *Bordetella *pneumonia associated with COVID-19.

Our patient had various risk factors for complicated SARS-CoV-2 infection [10]: he was obese, had diabetes mellitus, hypertension, history of renal transplantation, and was treated with immunosuppressants. Radiographic findings in COVID-19-associated pneumonia include bilateral, multifocal lung lesions, ground-glass opacities, consolidation, and pleural thickening [11]. Similar radiologic findings have been noted in *B. bronchiseptica-*associated pneumonia [3]. Infections with *B. bronchiseptica* in renal transplant patients have been uncommonly described. In one case, a patient with a history of renal transplant had bacteremia with *B. bronchiseptica* three weeks after undergoing a pancreas transplant who had exposure to dogs [12].

It is plausible to assume that our patient had an initial SARS-CoV-2 viral pneumonia, resulting in respiratory failure. *B. bronchiseptica* was cultured 12 days into his clinical course.

*B. bronchiseptica* infections in animals are usually treated with doxycycline, quinolones, or macrolides. Infections in humans have been treated with an anti-pseudomonal penicillins, quinolones, aminoglycosides, tetracycline, trimethoprim-sulfamethoxazole, or carbapenems [13]. Response to macrolides has been reported to be variable [14]. The frequency of macrolide resistance among *Bordetella *isolates in animals is low. In vitro experiments show that resistance to erythromycin develops quickly but that the organisms that developed resistance were unable to colonize mice, suggesting that the drugs may be effective in vivo [14].

Macrolides have not been the drugs of choice for treatment of *B. bronchiseptica* in humans, but it appears that susceptibility for this organism in vitro does not always predict clinical response [15]. It is therefore possible that this class of drugs may be effective in clinical settings, as happened in our case.

## Conclusions

Bacterial coinfection or superinfections should be considered in patients hospitalized with COVID-19-associated pneumonia when the clinical status deteriorates after initial improvement. Severe infection with rare bacterial organisms, especially in transplant recipients, is a concern. An accurate microbiological diagnosis is essential so that patient can be treated appropriately. Clinical response may not coincide with microbiologic in vitro susceptibility results.
